# Epidemiology of liver cancer in Kazakhstan: data from the Unified National Electronic Health System, 2014–2023

**DOI:** 10.1371/journal.pone.0330423

**Published:** 2025-08-21

**Authors:** Ayana Ablayeva, Dmitriy Syssoyev, Ruslan Akhmedullin, Altynay Beyembetova, Diyora Abdukhakimova, Temirgali Aimyshev, Iliyar Arupzhanov, Gulnur Zhakhina, Aigerim Biniyazova, Temirlan Seyil, Nurbek Igissin, Yuliya Semenova, Abduzhappar Gaipov

**Affiliations:** 1 Department of Medicine, Nazarbayev University School of Medicine, Astana, Kazakhstan; 2 Life Sciences and Health Research Institute, Ualikhanov University, Kokshetau, Kazakhstan; 3 Clinical Academic Department of Internal Medicine, Corporate Fund “University Medical Center”, Astana, Kazakhstan; Shiraz University of Medical Sciences, IRAN, ISLAMIC REPUBLIC OF

## Abstract

**Background:**

Liver cancer is the third-deadliest cancer globally, significantly affecting the Asian population, which accounts for 70% of cases. Despite these numbers, the burden of liver cancer in Central Asia, including Kazakhstan, remains underexplored. This paper reviews the epidemiology of liver cancer in Kazakhstan by analyzing large-scale administrative data from the national registry.

**Methods:**

The study cohort included 10,455 patients diagnosed with liver neoplasms (C22.0-C22.9, D13.4) from 2014 to 2023, using the International Classification of Diseases, 10th Revision (ICD-10). Age-standardized incidence, mortality, and prevalence rates per 100,000 population were calculated. Cox regression analysis estimated hazard ratios across social-demographic and medical predictors.

**Results:**

Over the decade, age-standardized incidence rates decreased from 5.55 to 5.40 per 100,000, while mortality rates rose from 3.75 to 4.75 per 100,000. A downward trend appeared between 2017–2019 but gradually reversed post-2020. Prevalence rates steadily increased from 1.30 to 7.32 per 100,000. The cohort’s mean age was 63.2 ± 0.1 years, with most cases diagnosed as stage III hepatocellular carcinoma. West Kazakhstan, East Kazakhstan, and Pavlodar were the most burdened regions. Liver cirrhosis history predicted worse survival (Hazard Ratio, HR = 1.14; 95% CI 1.06–1.22), while primary hypertension showed a protective effect (HR = 0.82; 95% CI 0.78–0.86).

**Conclusions:**

Liver cancer morbidity and mortality rates have risen in Kazakhstan over the past decade, with a slight decrease during the early COVID-19 pandemic. Patients are mostly diagnosed with stage III hepatocellular carcinoma. West Kazakhstan, East Kazakhstan, and Pavlodar are the most affected regions, requiring better management of high-risk groups. Poor survival was linked to liver cirrhosis. Further research on the role of rising metabolic liver diseases and treatment options in Kazakhstan is needed to better predict liver cancer prognosis.

## Introduction

Liver cancer is one of the most commonly diagnosed cancers at an advanced stage and ranks as the third-deadliest cancer worldwide [1]. Its risk factors include both infectious agents, such as hepatitis B and C viruses, as well as behavioral factors such as alcohol consumption, intravenous drug use, and smoking [2], making its preventive measures challenging. Nevertheless, recent statistics suggest the global advancement in liver cancer control in the last three decades has been driven by improved immunization rates against viral hepatitis and expanded screening of hepatocellular carcinoma. Still, the mortality rates have continued to rise in low and middle-income countries, indicating a disparity in access to effective preventive interventions and the need for equity-based initiatives that address region-specific aetiologies [3]. Notably, 2020 estimates from the Global Cancer Observatory (GLOBOCAN) indicate that 70% of the global burden attributable to liver cancer is concentrated in the Asian continent, highlighting the importance of enhanced strategic liver cancer control in this region [4].

Given the vast socio-economic diversity across Asia, the burden and management of liver cancer vary significantly between subregions. Among these, Central Asia stands out as a region where multiple risk factors and determinants of liver cancer interact in complex ways [5,6], but temporal liver cancer trends remain underexplored. The high prevalence of HBV and HCV [7], combined with substantial alcohol consumption [8] and the increasing prevalence of non-communicable diseases [9] in Central Asia, underscores the need for comprehensive epidemiological studies on liver cancer in this area to strategically lower the burden of disease in this region. Despite this, it is one of the least investigated Asian regions in terms of cancer epidemiology, both as a whole and for individual countries. Moreover, the definition of Central Asia varies across global cancer studies, with Caucasian and East Asian countries included or excluded from study to study and distorting regional estimates [5,10,11]. The World Bank definition of Central Asia includes only five countries: Kazakhstan, Kyrgyzstan, Uzbekistan, Tajikistan, and Turkmenistan.

Among these countries, Kazakhstan presents an intriguing case of the Central Asia region with its relatively strong socio-economic status and mixed ethnic population. Over the last decade, Kazakhstan has established itself as a regional leader in hepatitis prevention initiatives [12]. The government has been providing HBV vaccinations since 1998 and has offered free treatments for both hepatitis B and C since 2010. From 2012, the government also initiated free liver transplantation services [13]. However, in-depth epidemiological studies on liver cancer in Kazakhstan are limited. A recent study [14] indicated that the incidence of liver cancer decreased from 2005 but began to rise again in 2012. Nevertheless, the mortality rate and survival estimate for patients diagnosed with liver cancer remain understudied. The initiation of a hepatocellular carcinoma screening program in the country in 2013, followed by its suspension in 2018 due to ineffectiveness [14], underscores the need to evaluate the liver cancer epidemiology in this region even more. This study aims to assess the burden of liver cancer in Kazakhstan, using large-scale administrative data records from 2014 to 2023 from the Unified Electronic Healthcare System (UNEHS) [15,16]. The trends of morbidity and mortality over the decade were assessed, and social-demographic factors and comorbidities predicting the survival outcome of liver cancer were identified for the cohort.

## Methods and materials

### Study design and population

**Fig 1:**
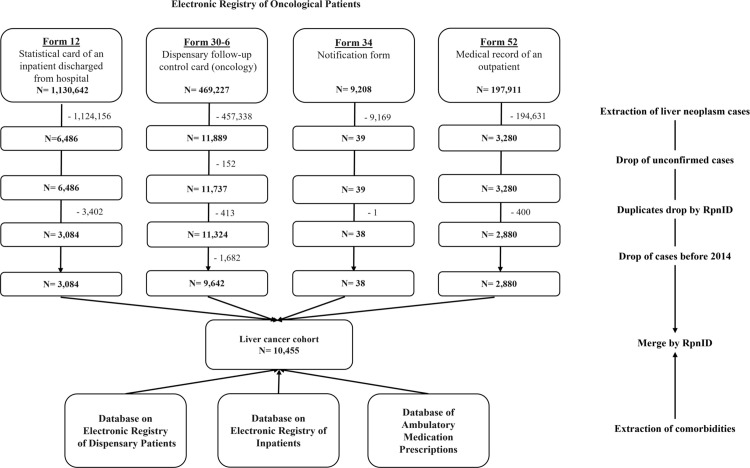
Data cleaning and preparation algorithm.

For this retrospective cohort study, data from 2014 to 2023 were obtained from the UNEHS [16] and accessed on November 9, 2024. Patients in the registry are assigned unique, lifelong population registry numbers (RpnIDs), ensuring that no identifiable information is available to identify individual participants during or after data collection. The study included patients diagnosed with liver neoplasms, identified using the International Classification of Diseases, Tenth Revision (ICD-10) codes as C22.0-C22.9 and D13.4, covering both benign and malignant liver neoplasms. The first record of the diagnosed primary oncological disease was retained. The algorithm of data cleaning is presented in the flow chart (Fig 1). The final cohort consisted of 10,455 patients. The data on population numbers was taken from the Bureau of National Statistics of Kazakhstan [17]. Additional information on the database used in this study can be found in S1 File.

### Exposure and covariates

Extracted data on the liver cancer cohort included variables on social-demographic information such as date of birth, date of death, sex, ethnicity, residence, region, and medical information such as cancer type and stage. The age was calculated by subtracting the date of birth from the date of diagnosis and afterwards categorized into five groups: (1) 0–29 years old, (2) 30–44 years old, (3) 45–59 years old, (4) 60–74 years old, (5) 75 and above years. Ethnicity contained more than 30 categories; therefore, to make data more manageable, the most prevalent ethnicities, such as (1) Kazakh and (2) Russian, were retained as separate groups, while less prevalent ethnicities were grouped into (3) Other category. Choropleth maps were constructed to compare average regional mortality and morbidity estimates for the study period. For this study, administrative structure of Kazakhstan before 2018 containing 2 administrative cities (Astana and Almaty) and 14 regions (Akmola, Aktobe, Almaty, Atyrau, East Kazakhstan, Jambyl, Karaganda, Kostanay, Kyzylorda, North Kazakhstan, Mangystau, Pavlodar, South Kazakhstan, West Kazakhstan) was used for map generation.

For the cancer type variable, cases with International Classification of Diseases for Oncology, 3rd Edition (ICD-O-3) morphology codes from 8170/3–8175/3 and ICD-O-3 topography code C22.0 were used to identify hepatocellular carcinoma. Cases with ICD-O-3 morphology code 8160/3 and ICD-O-3 topography code C22.1 were used for intrahepatic cholangiocarcinoma. The highest priority in classification was given to morphology, followed by a topology code. In the absence of these codes, ICD-10 codes were used. Due to the rarity of liver cancer cases coded as C22, C22.2-C22.9, they were grouped as other malignant liver neoplasms, while D13.4 cases were categorized as other benign liver neoplasms. Cancer stage variables contain stages from I-IV and the “unimplementable category” for benign neoplasms. Additional data on the comorbidities of patients, such as chronic hepatitis B, chronic hepatitis C, liver fibrosis, liver cirrhosis, diabetes, and primary hypertension, as well as death status, were separately extracted from other databases of UNEHS [15,18] and merged with the main dataset using RpnIDs. ICD-10 codes for comorbidities are given in S1 File.

The data on all social-demographic and some medical variables contained missing values. The pattern of missingness was investigated. Upon examination, it was concluded to perform complete-case analysis for all statistical analyses, as most missing values were found missing at random (MAR), except for age and sex, where missingness was very small to investigate its pattern. More details are given in the S1 File.

### Outcome assessment

New cases of liver cancer and deaths were found by the frequency of diagnosis and death events in each year. In 2014, total cases of liver cancer were computed by subtracting deaths from new cases; for subsequent years, total cases were derived by adding new cases to the previous year’s total and then subtracting that year’s deaths. Crude incidence and prevalence rates were determined by dividing new cases and total cases by the population of the corresponding year. All-cause and liver cancer-specific mortality rates were determined by dividing the total deaths and liver cancer-caused deaths, respectively, by the population for that year. Age-standardized rates for overall and sex-specific estimates were calculated using the standard population from the World Health Organization (WHO) [19]. The average annual percentage change (AAPC) for trends of age-standardized incidence, mortality and prevalence rates was determined. Mortality-to-incidence ratios (MIR) were calculated by division of death counts by incidence counts of the corresponding year. Rates specific to age, sex, residence, and region were calculated by dividing absolute numbers in each category by the respective population for each year. Due to the absence of population numbers for ethnicity categories for most of the period, morbidity and mortality estimates were not calculated for the subgroups of that classification. For the survival analysis, the start was the earliest date of liver cancer diagnosis, while the end date was either death or the end of follow-up, which concluded on December 31, 2023.

### Statistical analysis

Data was summarized in frequencies and percentiles for categorical variables and mean and standard deviation for continuous variables. Pearson’s Chi-square test was performed to investigate the relationship between groups of categorical variables and survival outcomes, and a two-sided t-test was used to see a difference between outcome groups in values of continuous variables. Incidence, prevalence, and mortality were presented as rates per 100,000 people. Mortality rates were also presented as per 1,000 person-years. Joint point regression analysis was performed to fit regression models and identify any significant changes in trends in the AAPCs. The weighted Bayesian information criterion was used for the determination of the best models. Overall and sex-specific age-standardized AAPCs and respective 95% confidence intervals for available data were evaluated. Cox regression analysis was performed to identify predictors of survival in the cohort [20]. The assumptions on proportionality of hazards were checked using Schoenfeld residuals and the log–log plot. As the benign subgroup of the predictor “stage” violated the proportionality of hazards, 276 observations in this category were dropped and omitted from the regression analysis. For other assumptions, observations were independent as each represented a unique individual with no repeated measures or clustering. There was also little chance of informative censoring before the death event, as follow-up of oncological patients was performed at the primary health-care level, and national death certificates were used to identify death or alive status. After all assumptions were verified, two predictive models were constructed and unadjusted and adjusted hazard ratios (HR) were estimated. A stepwise selection method was used for both models, and only predictors with a p-value of no less than 0.20 in bivariate analysis were used for model construction. Model 1 adjusted HR for social-demographic factors such as age, sex, ethnicity, and residence. Model 2 was adjusted for medical predictors in addition to the social-demographic factors. The fit of the model was assessed using Log-rank tests, the Akaike information criterion, and the Bayesian information criterion.

The significance level for all tests was set at 0.05. “STROBE” guidelines for observational studies were followed for all methods [21]. Statistical analysis was performed on STATA 16.1 MP2 version (STATA Corporation, College Station, TX) software. For jointpoint regression analysis, Joinpoint Trend Analysis Software was utilized (V5.2.0). The maps were created in Python (version 3.12.7; Python Software Foundation) using open-source libraries including GeoPandas for spatial data handling, Matplotlib for plotting, NumPy for numerical operations, and Shapely for geometric manipulations. A custom continuous color gradient was designed with Matplotlib’s LinearSegmentedColormap to visualize incidence rates across Kazakhstan’s regions. **The image template for maps were derived from open source database, Database of Global Administrative Areas (GADM).**

### Ethics statement

The study was approved by the Institutional Review Board of Nazarbayev University (protocol code: NU-IREC 651/24112022 and date of approval: 28 November 2022), with exemption from informed consent.

## Results

From 2014 to 2023, 10,455 patients were diagnosed with primary liver cancer, and 9,229 died, as detailed in Table 1. Males were 1.5 times more frequently diagnosed than females. The majority of patients (49.2%) were elderly individuals aged 60–74 years, with a mean diagnosis age of 63.2 ± 12.7 years. The least diagnosed age groups were 0–29 years and 30–44 years, collectively accounting for only 6% of the cohort. The cohort predominantly consisted of Kazakh people (65.6%), and a majority resided in urban areas (55.2%).

**Table 1:** Socio-demographic and medical characteristics of patients with liver cancer.

Demographic and medical characteristics	Total *n* = 10,455	Alive *n* = 1,226 (11.7%)	Dead *n* = 9,229 (88.3%)	p-value	Mortality rate per 1,000 PY [95% CI]
**Age, mean ± SD**	63.2 ± 12.7	56.3 ± 17.5	64.1 ± 11.7	**<0.001**	
**Age groups, *n (%)***				**<0.001**	
0-29	168 (1.6)	85 (6.9)	83 (0.9)		191.8 [154.6-237.8]
30-44	462 (4.4)	123 (10.0)	339 (3.7)		457.0 [410.8-508.3]
45-59	2,885 (27.6)	389 (31.7)	2,496 (27.0)		879.0 [845.2-914.2]
60-74	5,130 (49.1)	490 (40.0)	4,640 (50.3)		1,135.3 [1,103.1−1,168.4]
≥75	1,789 (17.1)	118 (9.6)	1,671 (18.1)		1,645.9 [1,568.9−1,726.8]
**Sex, *n (%)***				**<0.001**	
Female	4183 (40.0)	677 (55.2)	3,506 (38.0)		746.9 [722.6-772.1]
Male	6,247 (59.8)	524 (42.7)	5,723 (62.0)		1,296.9 [1,263.8−1,331.0]
**Ethnicity, *n (%)***				0.078	
Kazakh	6,775 (64.8)	782 (63.8)	5,993 (64.9)		997.0 [972.0−1,022.5]
Russian	2,085 (19.9)	204 (16.6)	1,881 (20.4)		1,200.7 [1,147.6− 1,256.2]
Other	1,467 (14.0)	168 (13.7)	1,299 (14.1)		1,025.8 [971.5−1,083.1]
**Residence, *n (%)***				**<0.001**	
Urban	5,767 (55.2)	663 (54.1)	5,104 (55.3)		1031.8 [1,003.9−1,060.6]
Rural	4,433 (42.4)	322 (26.3)	4,111 (44.5)		1,356.5 [1,315.6−1,398.6]
**Liver cancer type, *n (%)***				**<0.001**	
Hepatocellular carcinoma	8,332 (76.7)	823 (67.1)	7,509 (81.4)		1,191.8 [1,165.1−1,219.0]
Intrahepatic cholangiocarcinoma	671 (6.4)	71 (5.8)	600 (6.5)		1,244.3 [1,148.6−1,348.0]
Other malignant neoplasms	1,176 (11.2)	146 (11.9)	1,030 (11.2)		746.9 [702.7-794.0]
Benign neoplasms	276 (2.6)	186 (15.2)	90 (1.0)		83.2 [67.7-102.3]
**Liver cancer stage at the time of diagnosis,** ***n (%)***				**<0.001**	
I	226 (2.2)	105 (8.6)	121 (1.3)		257.6 [215.5-307.8]
II	1,552 (14.9)	321 (26.2)	1,231 (13.3)		582.4 [550.7-615.8]
III	5,707 (54.6)	405 (33.0)	5,302 (57.4)		1,393.3 [1,356.2−1,431.3]
IV	2,011 (19.2)	84 (6.8)	1,927 (20.9)		2,176.9 [2,081.8−2,276.3]
Benign neoplasms	276 (2.6)	186 (15.2)	90 (1.0)		83.2 [67.7-102.3]
**Comorbidities, *n (%)***					
Chronic hepatitis B	686 (6.6)	139 (11.3)	547 (5.9)	**<0.001**	610.4 [561.3-663.7]
Chronic hepatitis C	995 (9.5)	227 (18.5)	768 (8.3)	**<0.001**	637.7 [594.1-684.4]
Liver cirrhosis	1,263 (12.1)	134 (10.9)	1,129 (12.2)	0.188	970.1 [915.2−1,028.4]
Liver fibrosis	709 (6.8)	90 (7.3)	619 (6.7)	0.407	839.6 [776.0-908.4]
Diabetes	1,212 (11.6)	164 (13.4)	1,048 (11.4)	**0.038**	903.5 [850.4–959.9]
Hypertension	3,120 (29.8)	433 (35.3)	2,687 (29.1)	**<0.001**	806.8 [776.9-837.9]

Bold values indicate statistical significance, p < 0.05 based on two-sided t-test and Pearson’s Chi-square test.

A significant portion of patients were diagnosed with hepatocellular carcinoma (76.7%), followed by other types of malignant liver neoplasm (11.2%), intrahepatic cholangiocarcinoma (6.4%), and other benign liver neoplasms (2.6%). The primary diagnosis was most frequently identified at stage III. Concurrent diagnoses included chronic hepatitis B (6.6%), chronic hepatitis C (9.5%), liver cirrhosis (12.1%), liver fibrosis (6.8%), diabetes (11.6%), primary hypertension (29.8%)

### Incidence, prevalence, and mortality

**Fig 2:**
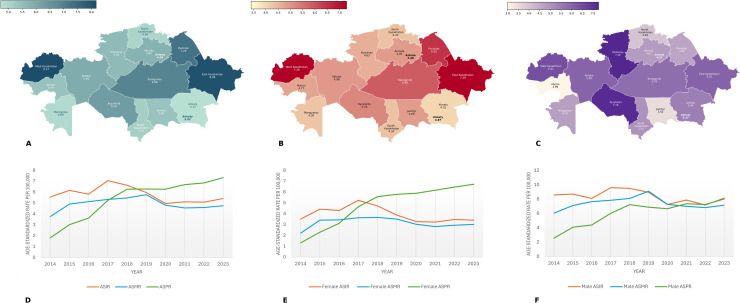
Regional and temporal burden of liver cancer in Kazakhstan (2014–2023). Regional average incidence **(A)**, mortality **(B)**, and prevalence (C) rates of liver cancer per 100,000 over 2014-2023. Age-standardized incidence, mortality, and prevalence rates of liver cancer per 100,000 over 2014-2023 for both sexes **(D)**, male-specific **(E)** and female-specific **(F)**. ASIR – age-standardized incidence rate; ASMR – age-standardized mortality rate; ASPR – age-standardized prevalence rate.

Overall, the age-standardized incidence rate (ASIR) decreased from 5.55 per 100,000 to 5.40 per 100,000 over a decade (Fig 2D and S1 Fig), but the change was found insignificant in the jointpoint regression model (AAPC −1.48; 95% CI −5.43–2.10). The trend exhibited fluctuations over the years, with the most notable increase occurring from 2016 to 2017. Following this peak, there was a gradual decline that persisted until 2020. Post-2020, the trend began a slow-paced increase. After stratification by sex, it was observed that ASIR decreased in both males (8.58 to 7.86 per 100,000) and females (from 3.50 to 3.40 per 100,000). Specifically, the decrease in ASIR from 2017 to 2023 was found statistically significant in females (S1 Fig). Across other social strata, the highest crude incidence rates over the period were among people over the age of 75 years and rural residents (S2 and S3 Fig). For both males and females, age at onset of disease peaked at the 70–74 years old group and declined thereafter, with a higher incidence prevailing in females after the 80–84 age group (S4 Fig). The incidence of hepatocellular carcinoma was the highest over the years (S5 Fig). The regions with the highest average incidence rates for the whole study period were West Kazakhstan (8.17 per 100,000), East Kazakhstan (8.08 per 100,000) and Pavlodar (7.29 per 100,000) regions (Fig 2A). The lowest incidence was in Astana city (4.24 per 100,000) and Almaty region (4.72 per 100,000).

The all-cause age-standardized mortality rate (ASMR) increased from 3.75 per 100,000 to 4.75 per 100,000 over a decade (Fig 2E). This change was also insignificant (AAPC −1.97; 95% CI −2.30–5.37) in the jointpoint regression model, but the increase in mortality rates from 2014 to 2016, and the decrease from 2016 to 2023 were found to be statistically significant (S1 Fig). The highest peak in the trend occurred in 2019, followed by a gradual decline till 2023. After stratification by sex, it was observed that ASMR increased both in males (6.03 to 7.14 per 100,000) and females (from 2.20 to 3.00 per 100,000). Across other social strata, the highest crude mortality rates over the period were among people over the age of 75 years and rural residents (S2 and S3 Fig). For both males and females, age at death peaked at the 70–74 years old group and declined thereafter, with higher mortality prevailing in females after the 80–84 age group (S4 Fig). Across regions, the highest average all-cause mortality rates from 2014 to 2023 were in East Kazakhstan (7.29 per 100,000), West Kazakhstan (7.26 per 100,000) and Pavlodar (6.50 per 100,000) regions (Fig 2B). The regions with the lowest mortality rates were Astana (3.40 per 100,000) and Almaty (3.87 per 100,000) cities. MIR values were high with an average value of 0.85 and increased from 0.67 to 0.88 over the study period (S6 Fig).

The age-standardized prevalence rate (ASPR) exhibited a gradual increase, rising from 1.80 per 100,000 in 2014 to 7.32 per 100,000 in 2023 (Fig 2F). The change was statistically significant in the jointpoint regression model (AAPC 16.22; 95% CI 13.63–19.22) (S1 Fig). The most significant increase occurred between 2016 and 2017, from 3.62 to 5.24. After stratification by sex, it was observed that ASPR increased both in males (2.55 to 8.01 per 100,000) and females (from 1.30 to 6.71 per 100,000). Across other social strata, the highest prevalence rates over the period were among people ≥75 years old and urban residents (S2 and S3 Fig). The highest average prevalence rates of liver cancer during the study period were in the Kostanay (7.26 per 100,000) and Kyzylorda (7.16 per 100,000) regions, while the smallest estimate was in the Atyrau region (2.99 per 100,000).

### Relationship of survival with social-demographic factors

Results of Cox regression analysis in Table 2 reveal that with increasing age, the risk of death substantially increases. After adjustment to social-demographic factors and medical predictors, the difference becomes even substantial for patients aged 30−44 (HR = 2.11; 95% CI 1.57–2.82), 45−49 (HR = 2.93; 95% CI 2.23 3.85, 60−74 (HR = 3.25; 95% CI 2.84–4.27), and ≥ 75 years (HR = 3.82; 95% CI 2.90–5.03) compared to patients aged 0−29 years. Male patients had a higher risk of all-cause death (unadjusted HR = 1.20; 95% CI 1.15–1.25) compared to female patients, which remained stable after the adjustment (adjusted HR = 1.18; 95% CI 1.13–1.26). Russians had a lower chance of survival than Kazakhs with an unadjusted HR of 1.26 (95% CI 1.19–1.33) and an adjusted HR of 1.18 (95% CI 1.12–1.26) in Model 2. Other ethnic representatives had a higher probability of death in a model adjusted to socio-demographic factors (HR = 1.12; 95% CI 1.16–1.27), but the association became insignificant after further adjustment to medical predictors (HR = 1.05; 95% CI 0.99–1.12). Rural residents demonstrated higher all-cause mortality in all models, with a 9% higher risk in Model 2 (HR = 1.09; 95% CI 1.05–1.14).

**Table 2:** Association between socio-demographic and medical parameters and all-cause mortality rates of liver cancer between 2014 and 2023.

Variable	Unadjusted HR [95% CI]	p-value	Adjusted to demographics HR [95% CI]	p-value	Adjusted to medical predictors HR [95% CI]	p-value
**Age groups, *n (%)***						
0-29	ref.		Ref.		Ref.	
30-44	**2.04 [1.52-2.73]**	**<0.001**	**1.93 [1.44-2.59]**	**<0.001**	**2.11 [1.57-2.82]**	**<0.001**
45-59	**2.72 [2.07-3.56]**	**<0.001**	**2.48 [1.89-3.26]**	**<0.001**	**2.93 [2.23-3.85]**	**<0.001**
60-74	**2.84 [2.17-3.73]**	**<0.001**	**2.64 [2.01-3.46]**	**<0.001**	**3.25 [2.48-4.27]**	**<0.001**
≥75	**3.38 [2.57-4.45]**	**<0.001**	**3.20 [2.44-4.22]**	**<0.001**	**3.82 [2.90-5.03]**	**<0.001**
**Sex, *n (%)***						
Female	ref.		Ref.			
Male	**1.20 [1.15-1.25]**	**<0.001**	**1.21 [1.16-1.27]**	**<0.001**	**1.18 [1.13-1.23]**	**<0.001**
**Ethnicity, *n (%)***						
Kazakh	ref.		Ref.		Ref.	
Russian	**1.26 [1.19-1.33]**	**<0.001**	**1.29 [1.22-1.37]**	**<0.001**	**1.18 [1.12-1.26]**	**<0.001**
Other	**1.09 [1.03-1.16]**	**0.004**	**1.12 [1.05-1.19]**	**<0.001**	1.05 [0.99-1.12]	0.109
**Residence, *n (%)***						
Urban	ref.		Ref.		Ref.	
Rural	**1.14 [1.09-1.19]**	**<0.001**	**1.18 [1.13-1.23]**	**<0.001**	**1.09 [1.05-1.14]**	**<0.001**
**Liver cancer stage, *n (%)***						
I	ref.		–	–	Ref.	
II	**1.90 [1.57-2.29]**	**<0.001**	–	–	**1.73 [1.43-2.08]**	**<0.001**
III	**3.36 [2.80-4.03]**	**<0.001**	–	–	**2.90 [2.42-3.48]**	**<0.001**
IV	**4.77 [3.96-5.74]**	**<0.001**	–	–	**4.00 [3.33-4.83]**	**<0.001**
**Comorbidities,** ***n (%)***						
Chronic hepatitis B	**0.60 [0.55-0.66]**	**<0.001**	–	–	**0.68 [0.62-0.74]**	**<0.001**
Chronic hepatitis C	**0.59 [0.55-0.64]**	**<0.001**	–	–	**0.65 [0.60-0.70]**	**<0.001**
Cirrhosis	**0.86 [0.80-0.91]**	**<0.001**	–	–	**1.14 [1.06-1.22]**	**<0.001**
Diabetes	**0.89 [0.83-0.95]**	**<0.001**	–	–	0.94 [0.88-1.01]	0.090
Primary hypertension	**0.80 [0.77-0.84]**	**<0.001**	–	–	**0.82 [0.78-0.86]**	**<0.001**

HR, Hazard Ratio. Bold values indicate statistical significance, p < 0.05 based on the Cox-proportional hazard model. Adjusted HRs account for other variables in the model.

### Relationship of survival with medical factors

The crude Cox regression analysis results show that as stage increases, the survival worsens for patients, with stage IV having the worst prognosis (HR 4.77; 95% CI 3.96–5.74) compared to stage I liver cancer (Table 2). The adjusted model showed that the history of cirrhosis was associated with 14% (HR = 1.14; 95% CI 1.06–1.22) lower survival in liver cancer patients. In contrast, the history of chronic hepatitis B was associated with 32% (HR = 0.68; 95% CI 0.62–0.74), chronic hepatitis C with 35% (HR = 0.65; 95% CI 0.60–0.70), and primary hypertension with 18% (HR = 0.78; 95% CI 0.62–0.74) higher survival in the cohort. Diabetes also showed 11% lower risk of death in the unadjusted model (HR = 0.89; 95% CI 0.83–0.95), but the relationship became insignificant in the adjusted model (HR = 0.94 (0.88–1.01).

## Discussion

The findings of our study showed that the prevalence of liver cancer in Kazakhstan increased from 2014−2023, aligning with global patterns [22]. Nevertheless, some fluctuations in incidence and mortality estimates from 2016 to 2017 and from 2019 to 2020 were characteristic of the regional trend, presumably due to the initiation and disruption of the screening program regionally and the onset of the COVID-19 pandemic globally. Additionally, survival estimates in a given liver cancer cohort were found to be better in patients with diagnosed chronic hepatitis B and C, and lower in patients with diagnosed liver cirrhosis after adjustments to social-demographic factors and comorbidities.

Our findings showed that Kazakhstani estimates were slightly higher than estimates from the Saudi Arabian study [23]. In contrast, both incidence and mortality rates of liver cancer were lower than estimates in China, Central Asia in the Global Burden of Disease (GBD) study from 2019 [24]. However, the Central Asia region in this GBD analysis encompassed Azerbaijan, Armenia, Georgia, and Mongolia. Notably, Mongolia, which bears the world’s highest liver cancer burden, skews regional estimates, which limits their applicability to specific countries like Kazakhstan. We also found that our estimates were the most similar to global ones from the 2019 GBD study [24], but lower than 2021 estimates from GLOBOCAN [11].

It is important to note that incidence estimates fluctuated over time, marked by a peak in 2017 and significant declines from 2015 to 2016 and again from 2017 to 2020. In contrast, the all-cause mortality rate peaked in 2019 and then gradually declined. Joint point regression analysis confirmed the statistical significance of AAPC in trends in the first half of the study period, but not for the second one, as it was limited by the number of datapoints to evaluate more than one joint point. The possible explanation for the decline in the incidence over the period can be attributed to different levels of secondary prevention of liver cancer over the decade. In Kazakhstan, according to the Cancer Care Development Program (2012−2016), the implementation of all existing national screening programs, including liver cancer, was not finalized until 2016, explaining small fluctuations between 2014–2016, and rapid improvement in the next year [25]. However, the suspension of this program in 2018 due to its low efficiency could lead to the gradual decrease observed in the incidence in post-2017 years [26]. The lowest incidence value in 2020 is likely explained by the pandemic, which disrupted cancer diagnosis globally due to reduced patient visits during lockdowns and limited diagnostic capacity as medical resources were redirected to COVID-19 patients [27]. It is estimated that at the beginning of the pandemic, the screening rate for hepatocellular cancer alone decreased by approximately 30–40% [27,28].

In contrast, it was expected that the all-cause mortality rate would increase in 2020 due to the excessive mortality the world experienced because of the COVID-19 pandemic onset, but estimates for this cohort showed the opposite trend. This could have occurred due to the lower number of cases in the cohort in 2020. In 2019, all-cause mortality peaked, particularly among the most at-risk groups aged 60–74 and those 75 years and older with MIRs of 0.96 and 1.11, respectively (S2 and S6 Fig). As a result, these high mortality rates combined in 2019 with the poor cancer diagnosis in 2020 could lead to a smaller liver cancer cohort in 2020, where death cases were proportionally smaller, with a small liver cancer cohort leading to a drop in all-cause mortality rate in that year.

Still annual mortality rate in the liver cancer cohort was high, as indicated by the high MIR values throughout the study period, showing that most patients died within the same year of diagnosis. This can be attributed to liver cancer in Kazakhstan being predominantly diagnosed at later stages, highlighting the need for new and more effective screening initiatives in the region. Although the effectiveness of previous screening programs was found to be low, it is noteworthy that the highest incidence of benign neoplasms peaked in the suspension year (S7 Fig).

The region-level analysis of average incidence and mortality estimates of liver cancer showed that West Kazakhstan, East Kazakhstan, and Pavlodar were the most burdened regions throughout the study period, which is consistent with the findings of another small local study [29]. The historic location of the Semipalatinsk nuclear test site in north-eastern Kazakhstan potentially explains this observation for the latter two regions, as high radiation levels in this geographical location are associated with an elevated risk of several solid cancers [30]. In case of West Kazakhstan, high liver cancer burden can be related to a high prevalence of hepatitis B and C viruses in this region [31], making its population more susceptible to liver cancer attributable to these factors.

The mean age in the cohort revealed liver cancer being predominantly diagnosed in the elderly population, corresponding with global trends. With the ascending age, survival also worsened due to cumulative exposure to risks of liver cancer over the years [32]. Moreover, hepatitis B and C vaccinations were introduced in Kazakhstan in 1998 and only with a birth dose [33]. It means the protective effect of this preventive measure in our cohort only covers patients aged 16 and younger in 2014 to those aged 25 and younger in 2023, making other and much older age groups at higher risk of liver cancer by one more predictor.

The males were found to be at higher risk of disease and mortality than females until the age of 80. The findings agree with the observed global and regional trends [14,22,34]. This disparity can be attributed to the different levels of exposure to liver cancer risk factors between males and females [35], while the reversed trend after 80 is due to survivorship bias.

In our cohort, Russian individuals exhibited a worse prognosis compared to Kazakh individuals. Subgroup analysis by ethnicity revealed no significant differences between Kazakhs and Russians in terms of other sociodemographic factors, cancer stage, or the presence of comorbidities covered in this study. This suggests that some unobserved effects of comorbidities, whether included or excluded from this study, might contribute to the disparity. This assumption is supported by another study, which observed that within a cohort of individuals diagnosed with chronic hepatitis B and C, Russian individuals had a higher risk of death compared to Kazakh individuals [34]. In addition, it is important to note that significant predictors of liver cancer outcomes, such as alcoholic and non-alcoholic liver diseases, were also omitted from the analysis due to the unavailability of data.

Rural residents have worse survival than urban people in our study, presumably due to differences in health behavior and availability of oncological medical services between these groups, observed globally [36,37]. Nevertheless, the lessened difference in incidence and mortality rates in recent years was also observed (S3 Fig) demonstrating positive outcomes in attempts of the government to bridge this gap through the introduction of telemedicine and mobile clinics in rural regions [38].

Among comorbidities, the presence of chronic hepatitis B or C showed a protective effect on the prognosis of liver cancer. This inference can reflect improved surveillance and care in these high-risk groups through initiatives of the Ministry of Health offering free screening and therapy options for the diagnosis and control of hepatitis diseases from 2010 and adopting a national roadmap in this matter in 2017 [12]. In our cohort, the proportion of patients diagnosed with stage II liver cancer was higher in the hepatitis-positive group, while stage IV liver cancer was more frequent in the hepatitis-negative group, supporting this assumption. However, it should be noted that patients with chronic hepatitis may be underrepresented in our cohort, and undiagnosed hepatitis could lead to a more severe progression and poor prognosis of liver cancer than diagnosed and treated hepatitis.

In contrast, patients with diagnosed liver cirrhosis had an elevated 14% mortality hazard ratio in the adjusted survival model. A study with a similar survival model construction shows liver cirrhosis worsens the prognosis of liver cancer by 45% and 47% in unadjusted and adjusted models, respectively [39], indicating that patients with cirrhosis can be left unnoticed in our cohort. The effect of cirrhosis alone on unadjusted models being associated with better survival models supports this assumption. It is observed that during the co-occurrence of liver cirrhosis and liver cancer, often the former goes unnoticed, but with exacerbation of liver inflammation and damage leading to a poorer prognosis than in recognized cases [40].

Diabetes showed a protective effect on survival, but the estimate was not statistically significant. The literature shows that diabetes is inversely associated with survival among liver patients due to exacerbation of inflammation of the liver, especially in interaction with other metabolic disorders such as non-alcoholic fatty liver disease (NAFLD) [41]. The absence of this relationship in our cohort could be due to the high underdiagnosis rates of diabetes in Kazakhstan [42].

The presence of hypertension showed better survival, contrasting findings from the literature [43]. However, similar relationships are observed in studies with other cancer types [44,45]. The explanation of this relationship is assumed to be rooted in the protective effects of antihypertensive medications. A recent study demonstrated that the intake of angiotensin receptor blockers and angiotensin-converting enzyme inhibitors showed 30% better survival among colorectal cancer patients compared to the control group [44]. The underdiagnosis of this comorbidity can also be another possible explanation for the observed positive relationship.

### Strengths and limitations

This study has several strengths. It is the first epidemiological research investigating the morbidity, mortality, and survival of liver cancer in a Central Asian country. Additionally, it utilizes a large administrative dataset, which bolsters its robustness. Nonetheless, the study also has its limitations. UNEHS registries contain information only on clinically important data, defined by hospitalisations, outpatient visits and official registration for medical follow-up. This implies that some liver cancer patients may be missing from the medical system database, and this study, if they have limited access to primary healthcare. In addition, due to insufficient data, the effect of other metabolic liver diseases, such as NAFLD or NASH, on the survival prognosis of liver cancer patients could not be estimated. Moreover, the registry data contains many missing values regarding the medical treatment of liver cancer, making estimation of their effect on survival also impossible. The results are specific to Kazakhstan for the period from 2014 to 2023 and may not be generalizable to other regions or time frames.

## Conclusions

Over the past decade, the morbidity and mortality rates of liver cancer have increased in Kazakhstan, with a slight decline observed during the early COVID-19 pandemic. Most patients are diagnosed at stage III with hepatocellular carcinoma. Advanced age over 75, male gender, rural residence, Russian ethnicity, and underlying liver cirrhosis were each associated with the poorest prognosis in their respective groups within this cohort. West Kazakhstan, East Kazakhstan, and Pavlodar regions should be prioritized as these regions share the biggest burden of liver cancer in the country. To further understand the underlying causes of disparities in liver cancer burden in the country, the impact of treatment outcomes and other potential factors and comorbidities as metabolic liver diseases, associated with disease progression and prognosis, should be thoroughly evaluated.

## Supporting information

S1 File Supplementary material. (DOCX)

S1 Fig Age-adjusted annual percent changes (APC) in incidence (A), mortality (B), and prevalence (C) rates per 100,000 over 2014–2023 for both sexes and sex-specific. (TIF)

S2 Fig Crude and age-specific incidence rates (A), all-cause mortality rates (B), and prevalence rates (C) of liver cancer per 100,000 over 2014–2023. (TIF)

S3 Fig Crude and residence-specific incidence rates (A), all-cause mortality rates (B), and prevalence rates (C) of liver cancer per 100,000 over 2014–2023. (TIF)

S4 Fig Age- and sex- specific median yearly incidence (A) and mortality (B) rate of liver cancer per 10⁶ population over 2014–2023. (TIF)

S5 Fig Crude and type-specific incidence rates (A), all-cause mortality rates (B), and prevalence rates (C) of liver cancer per 100,000 over 2014–2023. (TIF)

S6 Fig Crude and age-specific mortality to incidence ratios of liver cancer per 100,000 over 2014–2023. (TIF)

S7 Fig Crude and stage-specific incidence rate of liver cancer per 100,000 over 2014–2023. (TIF)

## Abbreviations

ASIR: age-standardized incidence rate
ASMR: age-standardized mortality rate
ASPR: age-standardized prevalence rate
AAPC: average annual percentage change
DALY: disability-adjusted life years
GBD: Global Burden Disease
GLOBOCAN: Global Cancer Observatory
HR: hazard Ratio
HBV: hepatitis B Virus
HCV: hepatitis C Virus
HCC: hepatocellular carcinoma
ICC: intrahepatic cholangiocarcinoma
ICD-10: International Classification of Diseases, 10th Revision
ICD-O-3: International Classification of Diseases for Oncology, 3rd Edition
MIR: mortality-to-incidence ratio
NAFLD: non-alcoholic fatty liver disease
NASH: non-alcoholic steatohepatitis
RpnID: population registry number
SDI: social-demographic index
UNEHS: Unified Electronic Healthcare System
WHO: World Health Organization
