# Supplementary material for: Epidemiology of liver cancer in Kazakhstan: data from the Unified National Electronic Health System, 2014–2023
Source: PLoS One. 2025 Aug 21;20(8):e0330423. doi: 10.1371/journal.pone.0330423 (PMC12370057; doi:10.1371/journal.pone.0330423)
Supplement: S1 File — (DOCX) [file pone.0330423.s001.docx]

**S1 Appendix. Database information**

In this study, primary data was sourced from the Electronic Registry of Oncological Patients of UNEHS. This registry encompasses comprehensive information on inpatients, outpatients, and dispensary patients diagnosed with oncological diseases in Kazakhstan, collected from various medical forms. These forms include:

- Form 012/у: Statistical card for inpatients discharged from the hospital.
- Form 030-6/y: Dispensary follow-up control card for oncology.
- Form 034/у: Notification form.
- Form 052/y: Medical report for outpatients.

The merged dataset from these forms contained missing values. For the liver cancer cohort, 0.2% of age data, 0.24% of sex data, 1.22% of ethnicity data, 5.36% of residence data, and 6.53% of stage data were missing. We examined whether the missingness pattern in variables such as age, sex, ethnicity, residence and stage was associated with other observed variables. For this purpose, the bivariate analysis using tests such as the Chi-square and Student’s t-test was conducted. In contrast, a more conservative method, like Little’s test, was not possible to perform because the amount of missing data was too low to meet the test’s degrees of freedom requirements. Results indicated that the missingness in ethnicity, residence and stage could be explained by variables as age and sex (S2-S4 Tables). However, for age and sex variables themselves, the missingness could not be demonstrated by observed data, as it was rare and overlapped with the missingness in other variables for any investigation test; only 9 and 11 observations were missing, respectively. Given the very low proportion of missingness, complete-case analysis was used for all statistical procedures, which was performed automatically via STATA software by excluding observations with missing values during bivariate and Cox regression analysis. Although the MAR assumption for this approach could not be fully verified for age and sex, the small amount of missing data suggests that any resulting bias or loss of power is likely negligible.

For the extraction of additional data on comorbidities, the Database on Electronic Registry of Dispensary Patients, Database on Electronic Registry of Inpatients, and Database of Ambulatory Medication Prescriptions of UNEHS were used. ICD-10 codes for extraction are given in Supplementary Table 1.

**S1 Table.** Extraction codes for comorbidities.

| **Comorbidity** | **ICD-10 codes** |
| --- | --- |
| Chronic hepatitis B | B18.0-B18.1 |
| Chronic hepatitis C | B18.2 |
| Cirrhosis | K74.3, K74.4, K74.5, K74.6 |
| Fibrosis | K74.0, K74.2 |
| Diabetes | E08-E11, E13 |
| Primary hypertension | I10 |

**S2 Table. Missingness pattern in the ethnicity variable.**

|  | **Ethnicity** | | |  |
| --- | --- | --- | --- | --- |
| **Other observed**  **variables** | **Total**  ***n* =10,455** | **Missing**  ***n* =128 (1.2%)** | **Present**  ***n* = 10,327 (98.8%)** | **p-value** |
| **Age, mean ± SD** | 63.2±12.7 | 29.1±28.7 | 63.5±12.0 | **<0.001** |
| **Sex, *n (%)*** |  |  |  | **0.02** |
| female | 4,183 (40.1) | 53 (41.4) | 4,130 (40.0) |  |
| male | 6,247 (59.9) | 50 (39.0) | 6,197 (60.0) |  |
| **Residence, *n (%)*** |  |  |  | **0.21** |
| urban | 5,767 (56.5) | 58 (45.3) | 5,709 (55.3) |  |
| rural | 4,433 (43.4) | 34 (26.6) | 4,399 (42.6) |  |
| **Stage, *n (%)*** |  |  |  | **<0.001** |
| I | 226 (2.3) | 6 (4.7) | 220 (2.1) |  |
| II | 1,552 (15.9) | 15 (11.7) | 1,537 (14.9) |  |
| III | 5,707 (58.4) | 19 (14.8) | 5,688 (55.1) |  |
| IV | 2,011 (20.6) | 24 (18.8) | 1,987 (19.2) |  |
| benign | 276 (2.8) | 29 (22.6) | 247 (2.4) |  |

**S3 Table. Missingness pattern in the residence variable.**

|  | **Residence** | | |  |
| --- | --- | --- | --- | --- |
| **Other observed**  **variables** | **Total**  ***n* =10,455** | **Missing**  ***n* =255 (2.4%)** | **Present**  ***n* = 10,200 (97.6%)** | **p-value** |
| **Age, mean ± SD** | 63.2±12.7 | 56.3±12.2 | 63.3±12.7 | **<0.001** |
| **Sex, *n (%)*** |  |  |  | **<0.001** |
| female | 4,183 (40.1) | 162 (63.5) | 4,021 (39.4) |  |
| male | 6,247 (59.9) | 70 (27.4) | 6,177 (60.6) |  |
| **Ethnicity, *n (%)*** |  |  |  | **0.001** |
| Kazakh | 6,775 (65.6) | 119 (46.7) | 6,656 (65.2) |  |
| Russian | 2,085 (20.2) | 63 (24.7) | 2,022 (19.8) |  |
| Other | 1,467 (14.2) | 37 (14.5) | 1,430 (14.0) |  |
| **Stage, *n (%)*** |  |  |  | **<0.001** |
| I | 226 (2.3) | 5 (2.0) | 221 (2.2) |  |
| II | 1,552 (15.9) | 17 (6.7) | 1,535 (15.0) |  |
| III | 5,707 (58.4) | 18 (7.0) | 5,689 (55.8) |  |
| IV | 2,011 (20.6) | 9 (3.5) | 2,002 (19.6) |  |
| benign | 276 (2.8) | 134 (52.5) | 142 (1.4) |  |

**S4 Table. Missingness pattern in the stage variable.**

|  | **Stage** | | |  |
| --- | --- | --- | --- | --- |
| **Other observed**  **variables** | **Total**  ***n* =10,455** | **Missing**  ***n* =683 (6.5%)** | **Present**  ***n* = 9,772 (93.5%)** | **p-value** |
| **Age, mean ± SD** | 63.2±12.7 | 62.1±14.9 | 63.2±12.6 | **0.03** |
| **Sex, *n (%)*** |  |  |  | **<0.001** |
| female | 4,183 (40.1) | 312 (45.7) | 3,871 (39.6) |  |
| male | 6,247 (59.9) | 359 (52.6) | 5,888 (60.2) |  |
| **Residence, *n (%)*** |  |  |  | **0.16** |
| urban | 5,767 (56.5) | 362 (53.0) | 5,405 (55.3) |  |
| rural | 4,433 (43.4) | 249 (36.4) | 4,184 (42.8) |  |
| **Ethnicity, *n (%)*** |  |  |  | **<0.001** |
| Kazakh | 6,775 (65.6) | 251 (36.7) | 6,524 (66.8) |  |
| Russian | 2,085 (20.2) | 304 (44.5) | 1,781 (18.2) |  |
| Other | 1,467 (14.2) | 93 (13.6) | 1,374 (14.1) |  |
