# Supplementary figures and images for: Epidemiology of liver cancer in Kazakhstan: data from the Unified National Electronic Health System, 2014–2023
Source: PLoS One. 2025 Aug 21;20(8):e0330423. doi: 10.1371/journal.pone.0330423 (PMC12370057; doi:10.1371/journal.pone.0330423)

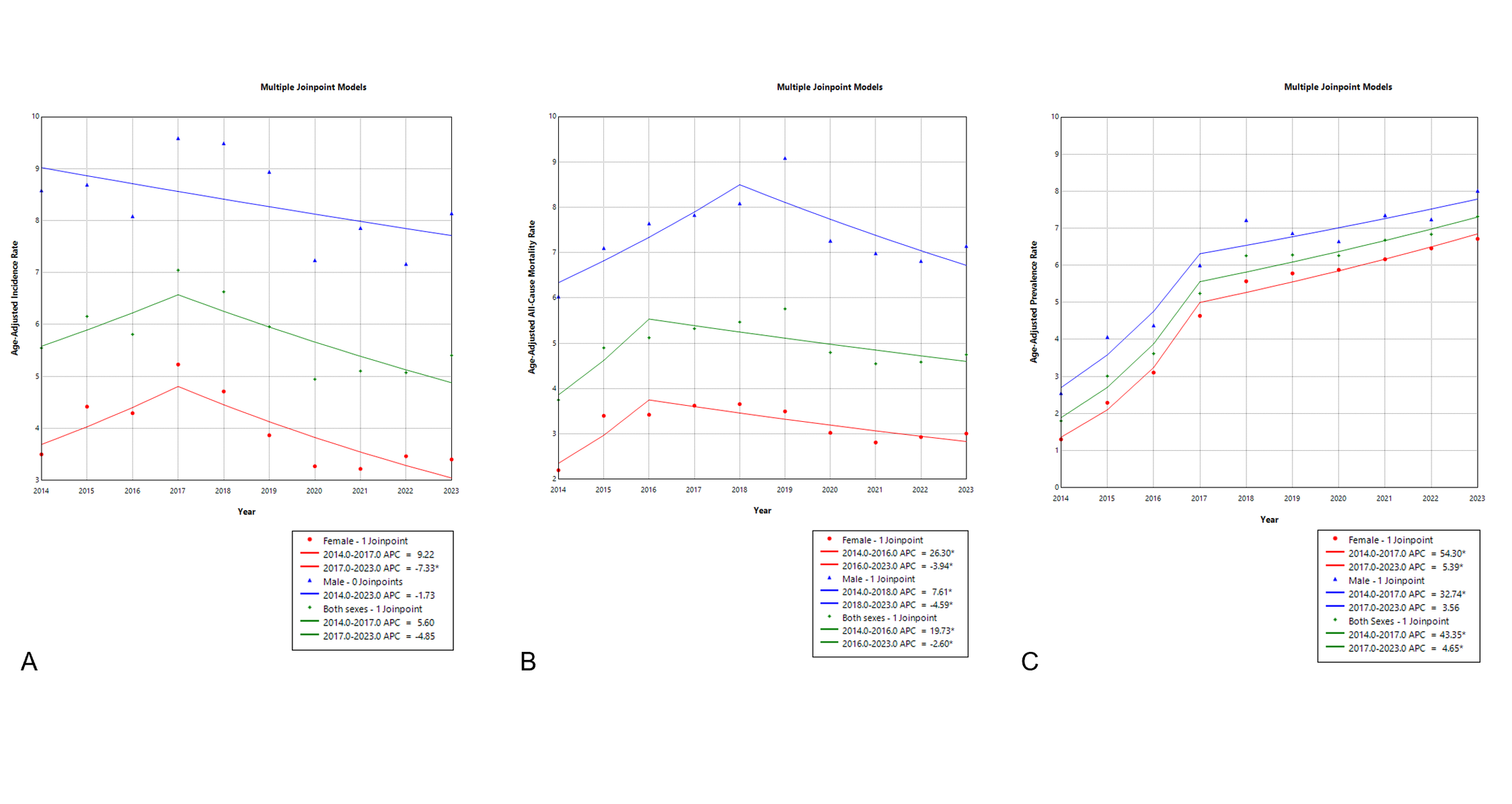

Supplement: S1 Fig — (TIF) [file pone.0330423.s002.tif]

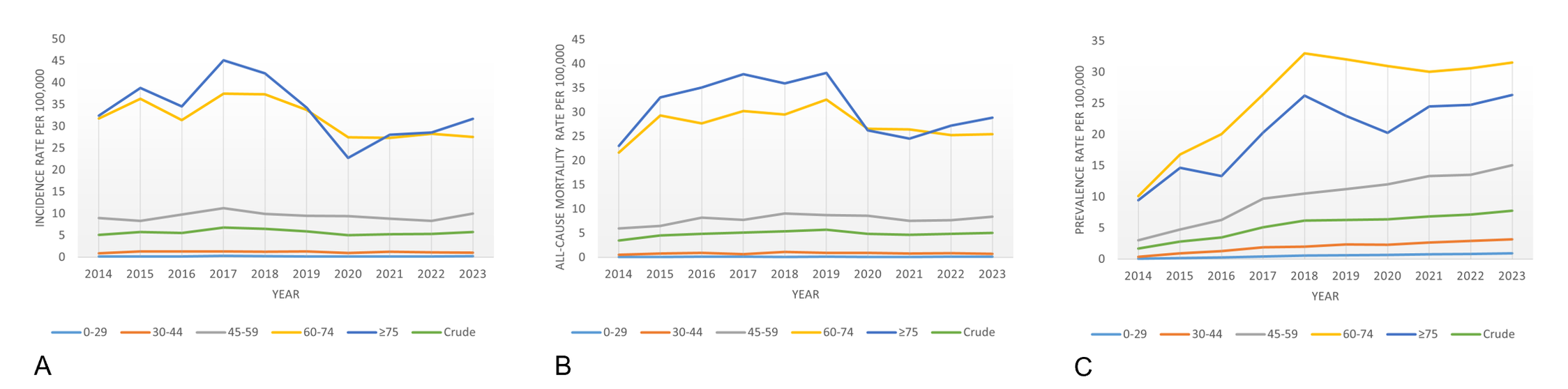

Supplement: S2 Fig — (TIF) [file pone.0330423.s003.tif]

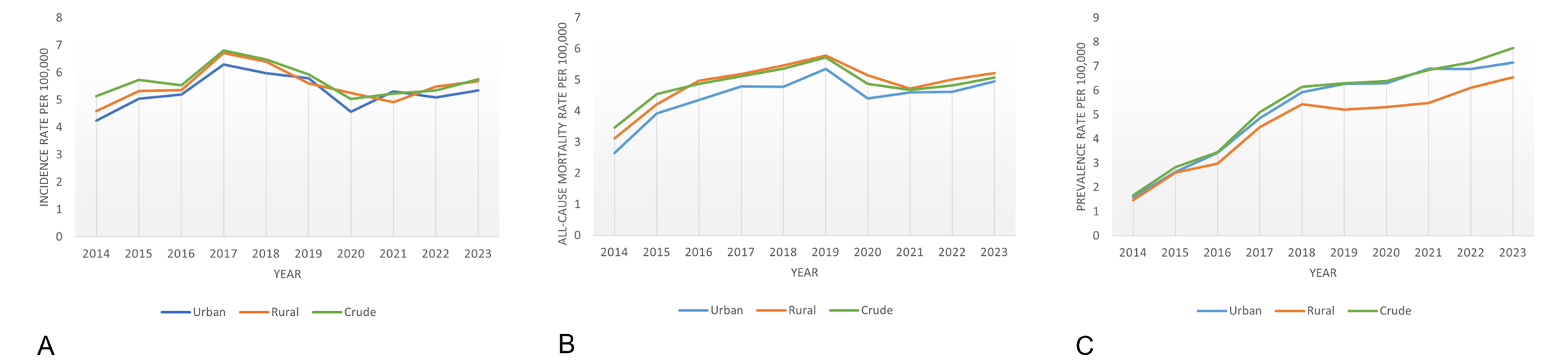

Supplement: S3 Fig — (TIF) [file pone.0330423.s004.tif]

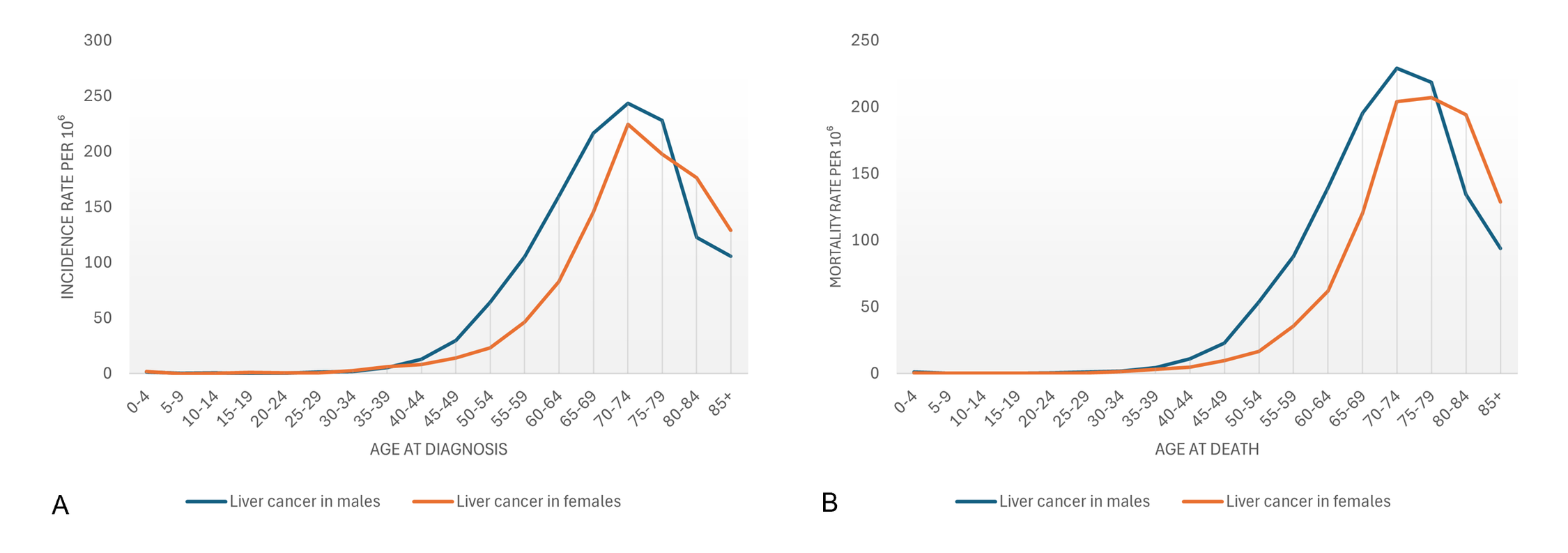

Supplement: S4 Fig — (TIF) [file pone.0330423.s005.tif]

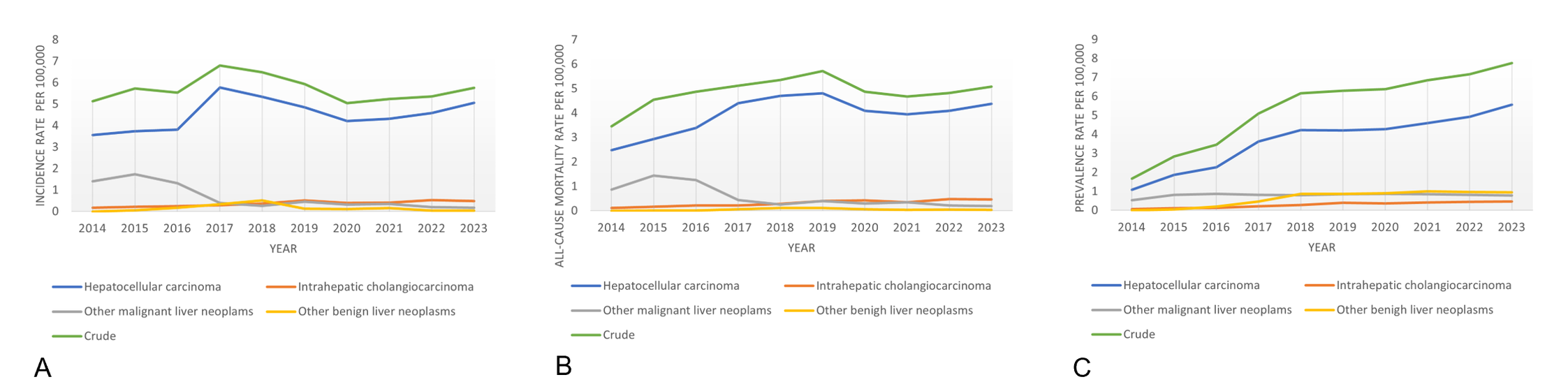

Supplement: S5 Fig — (TIF) [file pone.0330423.s006.tif]

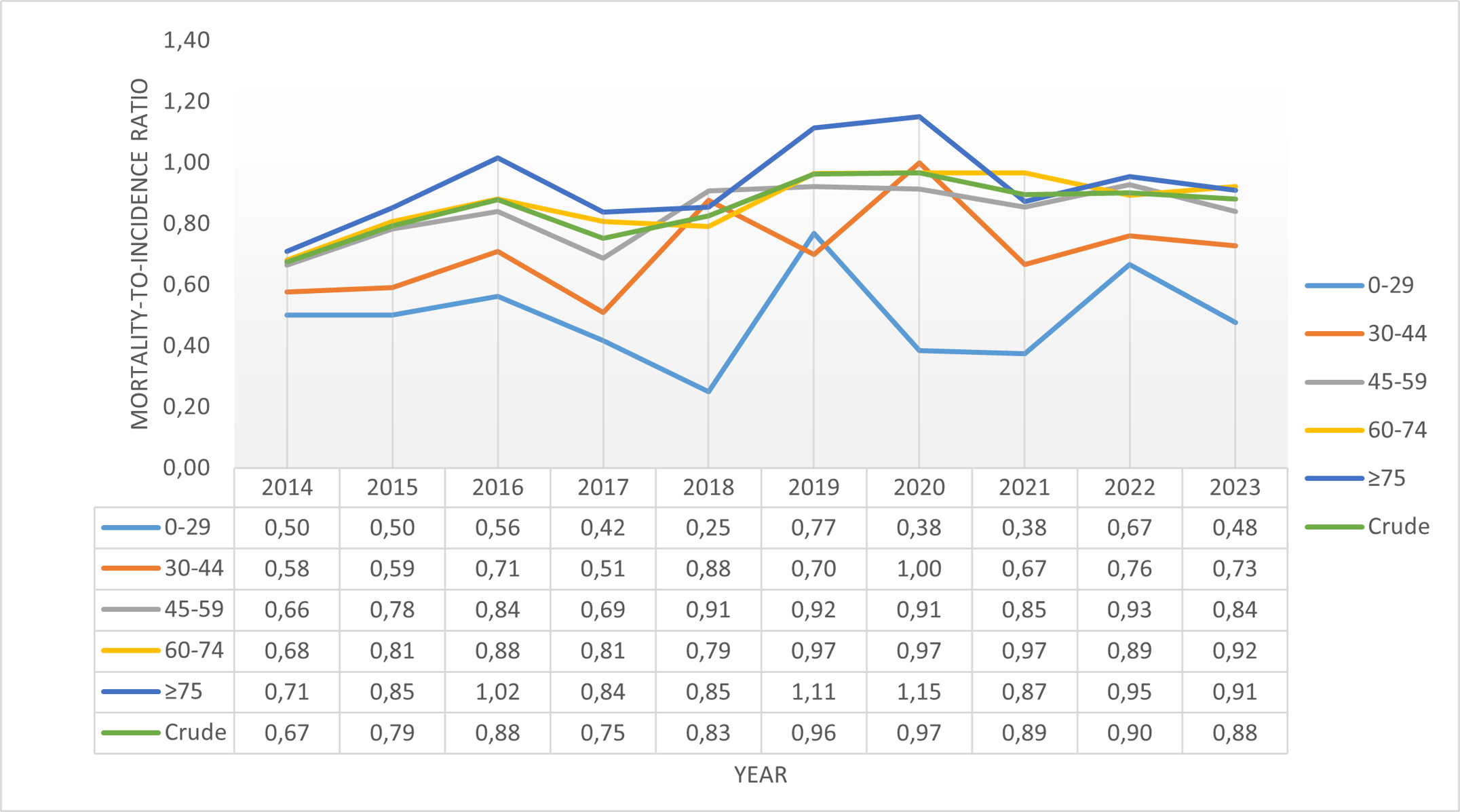

Supplement: S6 Fig — (TIF) [file pone.0330423.s007.tif]

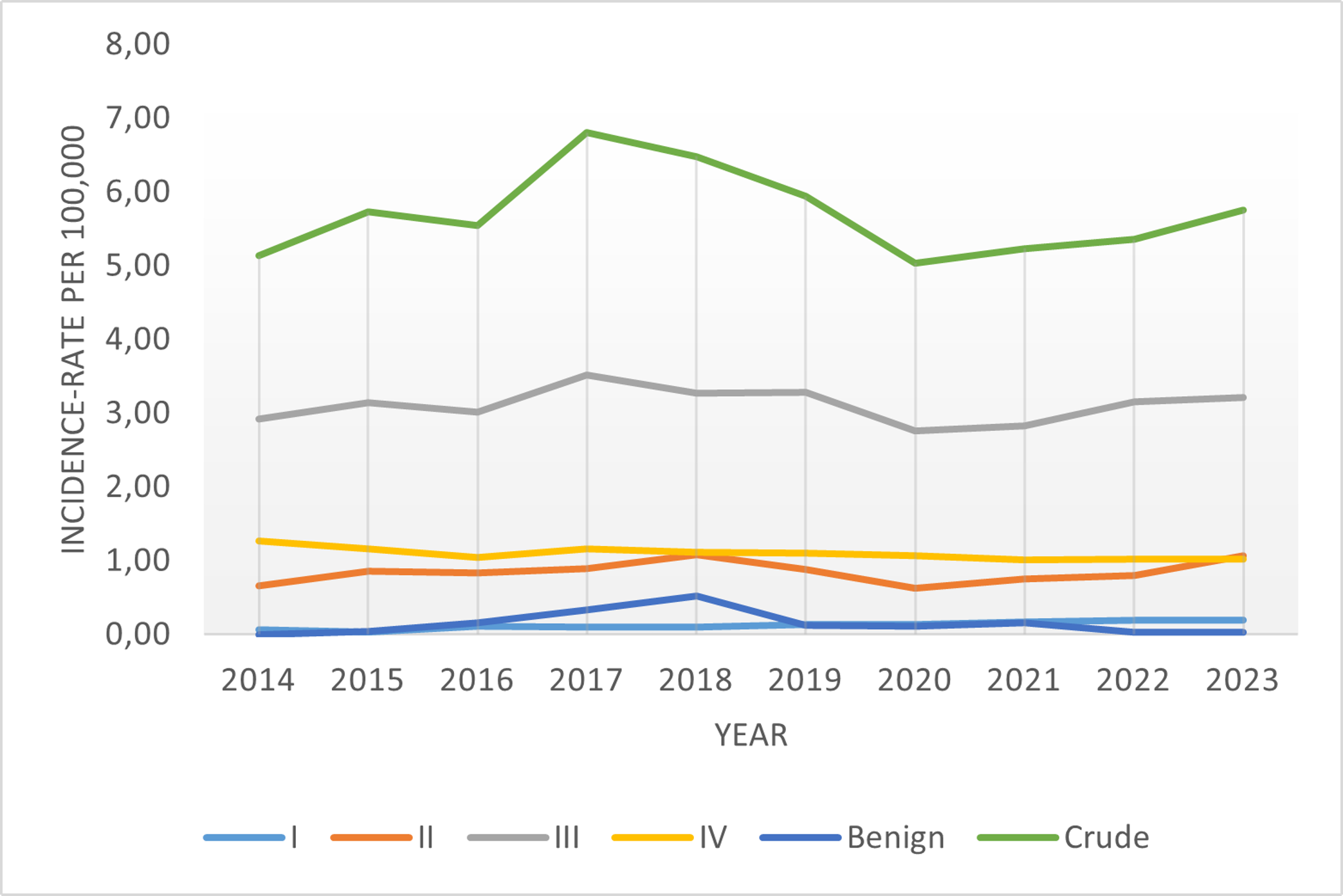

Supplement: S7 Fig — (TIF) [file pone.0330423.s008.tif]
